# Blood-Based Biomarkers of Neuroinflammation in Alzheimer’s Disease: A Central Role for Periphery?

**DOI:** 10.3390/diagnostics11091525

**Published:** 2021-08-24

**Authors:** Federica Angiulli, Elisa Conti, Chiara Paola Zoia, Fulvio Da Re, Ildebrando Appollonio, Carlo Ferrarese, Lucio Tremolizzo

**Affiliations:** 1 Neurobiology Laboratory, School of Medicine and Surgery, University of Milano-Bicocca, 20900 Monza, Italy; f.angiulli2@campus.unimib.it (F.A.); elisa.conti@unimib.it (E.C.); chiarapaola.zoia@unimib.it (C.P.Z.); ildebrando.appollonio@unimib.it (I.A.); carlo.ferrarese@unimib.it (C.F.); 2 Milan Center for Neuroscience (NeuroMI), 20126 Milano, Italy; 3 Ph.D. Program in Neuroscience, University of Milano-Bicocca, 20126 Milano, Italy; 4 Memory Clinic, Neurology Unit, ”San Gerardo” Hospital, 20900 Monza, Italy; f.dare@asst-monza.it

**Keywords:** Alzheimer’s disease, neuroinflammation, peripheral markers, monocytes, cytokines, chemokines, TSPO, delirium

## Abstract

Neuroinflammation represents a central feature in the development of Alzheimer’s disease (AD). The resident innate immune cells of the brain are the principal players in neuroinflammation, and their activation leads to a defensive response aimed at promoting β-amyloid (Aβ) clearance. However, it is now widely accepted that the peripheral immune system—by virtue of a dysfunctional blood–brain barrier (BBB)—is involved in the pathogenesis and progression of AD; microglial and astrocytic activation leads to the release of chemokines able to recruit peripheral immune cells into the central nervous system (CNS); at the same time, cytokines released by peripheral cells are able to cross the BBB and act upon glial cells, modifying their phenotype. To successfully fight this neurodegenerative disorder, accurate and sensitive biomarkers are required to be used for implementing an early diagnosis, monitoring the disease progression and treatment effectiveness. Interestingly, as a result of the bidirectional communication between the brain and the periphery, the blood compartment ends up reflecting several pathological changes occurring in the AD brain and can represent an accessible source for such biomarkers. In this review, we provide an overview on some of the most promising peripheral biomarkers of neuroinflammation, discussing their pathogenic role in AD.

## 1. Alzheimer’s Disease and the Quest for Biomarkers

Alzheimer’s disease (AD), widely recognized as the most common cause of dementia, is an age-related neurodegenerative disorder characterized by progressive cognitive deterioration, affecting both memory and other aspects of cognitive functioning. Age being the main risk factor for the disease, the marked increase in life expectancy is likely to determine a dramatic rise in the number of AD patients over the years, with substantial consequences on the public health worldwide [1]. The overwhelming impact that the increasing incidence of AD will have on global health can be mainly attributed to the inadequate and lacking understanding of its pathogenesis; the sporadic form of AD is influenced by various genetic and environmental factors and possesses a multifactorial etiology [2]. Despite being well-established that tau neurofibrillary tangles (NFT) and β-amyloid (Aβ) plaques are the hallmarks defining the neuropathology of this disease, it is not yet completely clear how much they contribute to neuronal degeneration spreading or if they may be just byproducts of ongoing damage [3]; this has been fully recognized and has led to the idea that other pathological processes can significantly contribute to AD pathogenesis as well [4]. Neuroinflammation, neuronal and synaptic degeneration, cerebral homeostasis disruption, metabolic disorders, vascular dysfunction and oxidative stress are just a few examples [5,6,7,8,9,10,11]. Furthermore, in spite of all the advances in modern medicine, a definitive diagnosis of AD is still based on the post-mortem detection of characteristic pathologic brain lesions—Aβ plaques and neurofibrillary tangles—similar to those described by Alzheimer himself in 1907 [12,13]. Additionally, the routine medical practice—based mostly on clinical and neuropsychological assessments in vivo—makes it possible to diagnose merely probable or possible AD [14]. Attempts have been made in the last few years to get to an in vivo neuropathological diagnosis of the disease, independent from the clinical symptoms [15]; however, the latest recommendation is to opt of a comprehensive approach, encompassing both the clinical assessment and in vivo neuropathological evaluation [16]. The goal is to diagnose AD as early as possible, in the prodromal phase (Mild Cognitive Impairment), when patients harbor pre-symptomatic pathological changes but maintain good brain functionality, in order to make use of treatments able to counteract or delay the appearance of symptoms [17]. As a matter of fact, therapies currently administered to patients with more advanced diseases are just symptomatic and, thus, unable to block the pathophysiological events leading to full-blown AD. Differently, the so-called “disease-modifying therapies” able to act on the cause and evolution of the disease by interfering with its pathogenesis are more effective when the brain damage is still restrained [18]. Under these circumstances, it is indisputable that, to successfully fight this neurodegenerative disorder, we require accurate and sensitive diagnostic and prognostic tools to be used for implementing an early diagnosis, the monitoring of disease progression and assessing the treatment effectiveness.

### 1.1. The Urgency for Diagnostic and Prognostic Biomarkers in Alzheimer’s Disease

Universally, the term biomarker refers to whatever feature that can be objectively measured and that is able to define the status of a biological entity at a given time [19]. The ideal biomarker for Alzheimer’s disease should closely reflect the succession of pathological events and prove potentially useful for a wide variety of applications. First and foremost, biomarkers are expected to be helpful for the early and differential diagnosis of AD. They should be able to reveal or suggest the existence of the fundamental alterations of the AD brain even during the preclinical asymptomatic phases of the disease; a better understanding of the biomarker signature preceding the clinical manifestation would enable the differentiation between the individuals at risk for AD and elderly people experiencing the normal aging process. At the same time, biomarkers should be able to correlate with the disease intensification—with the purpose of discerning between the different stages of AD pathology and predicting the conversion and progression to further cognitive decline—and to discriminate AD from other dementias. Furthermore, biomarkers should enable a more effective drug development in AD. They could have a role in the identification and screening of new therapeutic targets or potential therapeutic agents and in the monitoring of treatment efficacy and dosage, as well in clinical trials’ subject selections [20]. For the purpose of this review, the interest is focused on biological fluids-based biomarkers.

### 1.2. Blood-Based Biomarkers: Interplay between the Brain and the Periphery

Given its intimate contact with the central nervous system (CNS), the cerebrospinal fluid (CSF) represents a reasonable source for AD biomarkers. As a matter of fact, the CSF reflects the status of the CNS, and, therefore, the pathological changes in the brain—determining alterations in the levels of various substances—are able to affect its composition accordingly [21]. Both research and clinical practices have largely benefitted from biomarker discovery studies in the CSF over the years; on the one hand, those studies exceptionally contributed to the understanding of the natural history of AD [22]; on the other hand, the measurement of AD specific biomarkers (Aβ, Tau and pTau) in the CSF has been included in the criteria for the in vivo diagnosis of this disease [23]. However, the high invasiveness of CSF collection by lumbar puncture—together with the risks and costs associated with the procedure—has extensively limited its application in the standard clinical routine, given the reluctance of patients to undergo invasive and expensive testing repeatedly.

The limitations of CSF have prompted a switch of attention towards more accessible biological sources for biomarker research. The current focus is primarily on blood, whose collection is a routine procedure undeniably less invasive, inexpensive, replicable at regular intervals and easy to implement in large populations compared to lumbar puncture. As a consequence, blood-based biomarkers appear to be easily implementable in clinical practice to monitor the disease progression or treatment efficacy over time [24]. The rationale behind blood being a suitable source of biomarkers for this neurodegenerative disease lays in the evidence that the functionally impaired blood–brain barrier (BBB) of AD patients elicits an increased leakage of molecules from the CSF; those molecules end up reflecting the pathological changes occurring in the AD brain in the periphery, assuming the role of CNS informative blood biomarkers [25].

However, the analysis of blood biomarkers comes with an array of detection challenges, imputable to the nature of the sample and to the complexity of AD pathology, and it could often be difficult to correlate the changes in the CNS with those observed in the blood. First of all, the biomarkers’ concentrations are likely to be considerably low compared to the CSF, as they undergo substantial dilutions when entering into the bloodstream; in addition, given the pathological heterogeneity of AD, the BBB integrity can be affected differently, with consequences on the degree of crossing analytes. Moreover, brain proteins crossing the BBB may experience proteolysis in peripheral fluids. Secondly, it should be taken into account that AD can be accompanied by a systemic inflammatory response able to influence the blood levels of proteins indicative of the disease process; as a consequence, the fraction biomarkers attributable to the brain may be concealed by the one produced in the periphery. Finally, the blood owes a complex matrix—characterized by an abundant pool of antibodies and other molecules—that might interfere with the measurements of the biomarkers of interest, attenuating the sensitivity and specificity of the detection assays [24,26,27].

## 2. Neuroinflammation in the Pathogenesis of Alzheimer’s Disease

The term neuroinflammation refers to the broad range of inflammatory responses that originate in the CNS secondary to insults, injury or disease. Neuroinflammation has been described in AD since the first characterization of the disorder [12] and is now well-recognized as a central feature in its development, contributing to the pathogenesis just as much as the pathological hallmarks [4]. The pathological accumulation of Aβ and NFT in the brain is considered the principal trigger for neuroinflammatory responses in AD, inducing the activation of resident glial cells [28]. In any case, it should be noted that neuroinflammation priming is not a process specific for AD, since other protein aggregates (α-synuclein, TDP-43, etc.; collectively known as DAMP (danger-associated molecular patterns) and recognized by Toll-like receptors) are able to start such a response.

### 2.1. Microglia

Microglia are the resident innate immune cells of the CNS. They are ubiquitously distributed in the brain and act as the first line of defense, playing a fundamental role in its surveillance [29]. In AD, the microglia are able to identify and bind Aβ—through a range of different receptors—and become activated [30]. When activated, microglia go through morphological changes, localize in the vicinity of senile plaques and start releasing inflammatory mediators [5]. It should be noted that microglial activation is a complex process, producing multiple phenotypes according to the evolution of the inflammatory response, likely to have either beneficial or detrimental roles and effects. The classical (M1) form of microglial activation is characterized by the elevated production of proinflammatory cytokines—including tumor necrosis factor-alpha (TNF-α); interleukins IL-1β, IL-6, IL-12 and IL-18; interferon gamma (IFN-γ); chemokines like the monocyte chemotactic protein 1 (MCP-1) and neurotoxic agents—and is accompanied by impaired phagocytic capacity. On the other hand, the alternative (M2) activation phenotype is characterized by the release of several anti-inflammatory cytokines—interleukins IL-4, IL-10, IL-13 and transforming growth factor-beta (TGF-β)—tissue repair promotion and enhanced phagocytic ability [31,32]. Throughout AD progression, microglia switch from the M2 to M1 activation phenotype. The immediate activation of microglia in AD leads to a defensive response (acute inflammatory response) aimed at promoting Aβ clearance, thereby reducing its accumulation [33]. However, the long-standing exposure to Aβ—due to its increased production and accumulation over time—and inflammatory mediators (a result of the proinflammatory environment) induce a functional impairment in the microglia, whose phagocytic capacity becomes largely insufficient [34]. The failure of microglial phagocytosis increases the amyloid burden in the brain and facilitates direct Aβ-induced neurotoxicity [35]. During its turn, neuronal damage contributes to the release of further proinflammatory cytokines by activated microglia. This long-standing and self-perpetuating (chronic) neuroinflammatory response is often detrimental, resulting in neurodegeneration and brain function impairment [36].

### 2.2. Astrocytes

Astrocytes are the most abundant glial cells within the brain, part of the resident innate immune system of the CNS and active players in the neuroinflammatory response [37]. Astrocytes constitute a structural part of the BBB, with their processes make extensive contact with—and providing an almost complete coverage of—brain microvessels [38]. In AD, astrocytes are intimately associated with Aβ deposits, with their processes surrounding and even penetrating into the plaques to isolate the neighboring healthy tissue. Astrocytes accumulating around senile plaques are markedly hypertrophic and reactive, showing a striking upregulation of the glial fibrillary acidic protein (GFAP) [39,40]. Reactive astrocytes are involved in Aβ clearance across the BBB through Aquaporin 4 (AQP4) [41] and degradation [42]. Upon exposure to Aβ, reactive astrocytes release proinflammatory cytokines (mainly IFN-γ, IL-1β, IL6, TNFα and TGFβ) and upregulate the production of reactive oxygen species (ROS) and nitric oxide (NO) [43]; those mediators act either in an autocrine and a paracrine way, modulating the microglial activation [44] and self-perpetuating reactive gliosis. The persistent (chronic) activation of astrocytes results in atrophy and degeneration, decreased end feet coverage of cerebral microvessels and altered AQP4 perivascular localization [45]; as a consequence, Aβ clearance through the BBB is compromised, with a subsequent increase of the Aβ burden in the brain [46].

### 2.3. BBB Integrity and Permeability Are Compromised in AD Pathology

Proinflammatory mediators released by chronically activated glial cells in the AD brain could compromise the integrity and permeability of the BBB [47]. The BBB comprises various elements, collectively known as the neurovascular unit (NVU), including—besides endothelial cells—pericytes, astrocytes and oligodendrocytes [48]. The neuroinflammation and infiltration of toxic aggregates lead to the dysfunction of these components, resulting in a homeostatic imbalance conceivably playing a role in the progression of AD [49]. The reduction of pericytes has also already been demonstrated in AD [50,51], together with the CSF increase of the soluble marker of pericytes damage, PDGFR-β [52]. Oligodendrocytes are vulnerable to neuroinflammation and Aβ oligomers, and the resulting BBB dysfunction and myelin breakdown further amplifies the AD-related damage [53]. Furthermore, besides their role in maintaining the integrity of the NVU/BBB, oligodendrocyte progenitor cells may be a source of further Aβ secretion [54]. Under physiological conditions, the BBB completely separates the CNS from the peripheral immune system [55]; however, proinflammatory cytokines, chemokines, NO, ROS and metalloproteinases (MMPs) are able to increase the BBB permeability, ultimately leading to the withdrawal of the immune privilege of the brain. In particular, proinflammatory cytokines and oxidative stress modulate the BBB permeability by acting on the tight junctions in the cerebrovascular endothelial cells [56,57], while MMPs digest the endothelial basal lamina necessary for BBB integrity [58]. A major contributor to this process is also cerebral amyloid angiopathy (CAA), a condition characterized by the accumulation of Aβ in the cerebrovasculature; amyloid-laden vessels—often associated with degenerated smooth muscle cells, pericytes and endothelial cells—show reduced integrity, with implications for BBB permeability [59].

## 3. Involvement of Peripheral Immune System in the Development of Alzheimer’s Disease

Neuroinflammation was originally defined as a CNS-limited process; however, evidence increasingly shows that the peripheral immune system is involved in the pathogenesis and progression of AD. This may be counterintuitive at first, since AD is not a “classic” inflammatory disorder. Nevertheless, the existence of an immune–brain axis (see below, Section 3.2.1), the mechanism of inflammaging (Section 3.2.2) and the two-way relationship with delirium (Section 3.2.3) are just some of the evidence that changed our perception about a potential “peripheral” contribution to a central process. As aforementioned, microglial and astrocytic activation leads to the release of soluble inflammatory mediators that, despite being released locally, are able to cross the BBB, diffuse into the bloodstream and migrate to the periphery, thereby recruiting peripheral immune cells [60]. At the same time, the CNS is particularly responsive to peripheral immune system activation; proinflammatory cytokines—released by peripheral cells in response to pathogens and injury—are transported by the blood flow towards the BBB; here, they cross into the brain parenchyma and act upon glial cells, increasing their susceptibility to disease [61].

### 3.1. Peripheral Immune Cells Come to the Rescue of Functionally Impaired Glial Cells

#### 3.1.1. Monocytes/Macrophages

Circulating monocytes are predominantly recruited in the AD brain in a CCR2/CCL2-dependent manner. CCR2 is a monocytic surface receptor for CCL2 (also known as MCP-1), one of the most effective chemotactic factors for monocytes, upregulated near Aβ deposits as a result of the release from plaque-associated microglia [62]. Noteworthy, the process can be potentiated by the concurrent intervention of soluble Aβ, proven to be a powerful chemotactic stimulus both in vitro blood–brain barrier models and in AD transgenic mice [60,63]. In addition, the translocator protein 18 kDa (TSPO) may give an additional contribution; indeed, previous ex vivo human studies have demonstrated both the presence of functional TSPO receptors on monocyte surfaces [64] and the ability of various TSPO ligands to influence their chemotaxis [65].

The contribution of circulating monocytes to AD pathogenesis is still controversial. In vivo studies using mouse models of AD showed that the recruited monocytes are able to cross the BBB in areas of greater permeability, reach the perivascular space and, from there, infiltrate the brain parenchyma, associating with the regions of increased Aβ deposition [66]. Upon recognition of the Aβ peptides, infiltrating monocytes appear to differentiate into macrophages and to contribute to the clearance of Aβ from both the brain vasculature and parenchyma, thereby restricting Aβ plaques and reducing the degree of CAA [63,67]. On the other hand, monocytes isolated from AD patients exhibit reduced differentiation into macrophages—together with decreased phagocytosis and the lysosomal degradation of Aβ—compared to monocytes from age-matched healthy controls [68].

#### 3.1.2. Lymphocytes

The role of the adaptive immune system in AD is far from completely elucidated; however, the ablation of T- and B-lymphocytes results in a significant acceleration of amyloid pathogenesis and worsening of neuroinflammation [69]. A number of reports have confirmed an increase of CD4+ and CD8+ T cells in the brains, blood and CSF of AD patients [70,71]. In addition, the number of B cells was found significantly increased in the blood of AD patients compared to healthy controls (given their role in antibody secretion, direct tissue infiltration is not necessarily required) [72]. T cells can interact with microglia and modulate their phagocytic and secretory phenotypes, either by infiltrating the brain through the damaged BBB or exerting their function from the periphery (via cytokine secretion) [73]. On the other hand, B cells can be induced by Th2 modulation to produce immunoglobulins—and, among them, anti-Aβ antibodies. Anti-Aβ antibodies have proven able to promote Aβ clearance, thereby reducing its deposition in plaques via different mechanisms; they can bind Aβ in the brain and favor its degradation by microglial cells [74] or facilitate its efflux through the BBB [75]; alternatively, they can sequester Aβ in the peripheral blood, lowering its free level, and induce its release from the brain (peripheral sink effect) [76]. Furthermore, these naturally occurring anti-Aβ auto-antibodies (NAb) may have a role in preventing Aβ toxicity and aggregation [77,78].

### 3.2. Systemic Inflammation Enhances CNS Susceptibility to Disease

#### 3.2.1. Immune–Brain Axis

Circulating proinflammatory cytokines, released by peripheral immune cells in response to pathogens and injury, are able to communicate with the CNS by crossing into the brain parenchyma—either passively, through leaky areas of the barrier, or by active transport systems (receptors on epithelial cells) [79]; alternatively, they can stimulate the afferent vagus nerve to induce signaling within the brain [80]. This increased inflammatory signaling within the brain is able to act upon the resident immune cells, promoting their activation and shift towards a proinflammatory phenotype. A single challenge induces only a transient activation, with the glial cells returning to their normal resting state shortly thereafter [81].

Interestingly, an increasing amount of data indicate that the gut microbiota may play a major role in priming “neuro”-inflammation and oxidative stress [82]. Furthermore, psychoactive substance release and decreased levels of neurotrophic metabolites have also been related to altered microbiota compositions and may participate in AD pathogenesis as well, besides potentially inducing direct cognitive and behavioral effects [83]. In particular, immune cells are deeply involved in regulating the gut microbiota homeostasis, and significant changes in the host mononucleate phenotype result from the exposure to the vast array of soluble mediators released in the gut (microbiota-related TLR ligands and cytokines, mainly) [84]. These changes participate in AD pathogenesis, promoting an inflammatory “inclination” that is presumably part of the process known as “inflammaging” (see below) [85]. Thus, the possibility of targeting the gut microbiota is nowadays considered as promising and has led to the development of nutritional interventions, besides microbiota transfer, which has also been shown in experimental animal models to reduce AD-related pathology [86].

#### 3.2.2. Inflammaging and Glial Priming

Throughout the aging process, the longstanding exposure to internal and external damaging agents promotes a chronic low-grade activation in the immune system, with a consequent increase in its proinflammatory status (inflammaging) [87]. Likewise, the resident immune cells of the brain undergo a progressive shift toward a proinflammatory activated state with aging; the persistent stimulation over time makes them hypervigilant, enhancing their sensitivity and response to inflammatory stimuli (priming) [88]. As a consequence, a certain degree of neuroinflammation would already be detectable in the prodromal phase of AD, even before Aβ and NFT deposition [89].

#### 3.2.3. Sickness Behavior and Delirium

The existence of an intercommunication between the brain and the periphery is clearly supported by the occurrence of behavioral symptoms—a syndrome known as sickness behavior—in response to proinflammatory cytokines released in the framework of systemic inflammation [90]. It is now clear that the same systemic inflammatory signals can have severe deleterious effects on brain functions when occurring in old age or in the presence of a preexisting neuronal vulnerability. Delirium represents an extreme form of sickness behavior characterized by disturbances in multiple aspects of cognitive functioning ascribable to neuronal and synaptic dysfunctions [91]. These symptoms allegedly reflect an exaggerated response of the CNS to systemic inflammatory stimuli due to the microglial priming [92] archetypal of advanced age and dementia, indeed recognized as the most common risk factors for delirium [93,94]. At the same time, delirium can affect the course of dementia by exacerbating cognitive impairment [95]. It is now recognized that delirium and dementia share overlapping clinical features and common pathogenic mechanisms, to the point that it is possible to speculate that both disorders represent different stages of a common process [96].

#### 3.2.4. The Cholinergic Anti-Inflammatory Pathway

**Figure 1:**
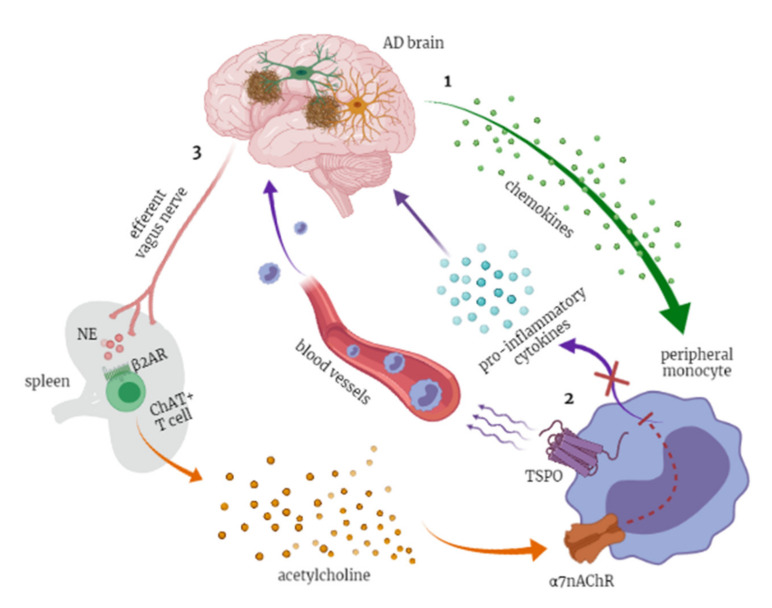
(1) Beta-amyloid oligomers and their subsequent deposition in plaque fuel neuroinflammation, leading microglia to produce chemokines that lure monocytes into the brain. The chemotaxis process is regulated, among other players, by the TSPO, expressed by monocytes and well-known markers of activated microglia. (2) Besides entering the blood–brain barrier, peripheral monocytes produce proinflammatory cytokines that represent the afferent arm of the “inflammatory reflex”. (3) The efferent arm of this reflex is known as the “cholinergic anti-inflammatory pathway” (CAIP) and is represented by a vagal efflux indirectly stimulating the activation of the α7-nicotinic cholinergic receptor (α 7nAChR) expressed by peripheral monocytes and shutting off inflammasome and cytokine production. Created with BioRender.com.

Persistent inflammatory stimuli could result in chronic inflammation, a common risk factor for AD, if immune homeostasis is not restored. In this regard, it has been demonstrated that the CNS is able to monitor and regulate inflammatory responses in real time by means of the cholinergic anti-inflammatory pathway (CAIP) [97]. The so-called inflammatory reflex is triggered when peripheral inflammatory stimuli signal the nucleus tractus solitarius (NTS) by interacting with their receptors on the vagus nerve afferent fibers; once processed in the brain, the outbound signals are transmitted to the spleen via the efferent vagal nerve and splenic nerve. Upon activation, the splenic nerve releases norepinephrine (NE), which interacts with β2-adrenergic receptors expressed on choline acetyltransferase (ChAT)-positive T cells in the spleen, increasing acetylcholine (ACh) synthesis and release. Finally, the binding of ACh to α7 nicotinic acetylcholine receptors (α7nAChRs) expressed on macrophages inhibits proinflammatory cytokine synthesis and release, resulting in alleviated systemic inflammation [98] (Figure 1). Pharmacological intervention on the cholinergic anti-inflammatory pathway is a real possibility; for one, the acetylcholinesterase inhibitor Donepezil is able to act on α7nAChR expressed by peripheral blood mononuclear cells (PBMC) and mediate an immunomodulatory response [78].

## 4. Blood-Based Biomarkers of Neuroinflammation

The evidence outlined above of an involvement of the peripheral immune system in AD pathogenesis inevitably offers the possibility of a theoretical approach to the blood compartment as a source of potential biomarkers. A selection of such targets is provided in this section, although, at the moment, none of them has a tangible clinical use. This may be mainly due to the fact that these markers of immune system perturbation may indeed change in the blood following inflammatory events other than AD. Nevertheless, these markers represent very interesting opportunities for a better understanding of AD pathogenesis, and in the future, they may form part of the panels for precision medicine, identifying selected patients for targeting drugs.

### 4.1. Cytokines as Biomarkers of Immune System Activation

Cytokines released by immunocompetent cells—whether they are peripheral immune cells or resident glial cells—promote the bidirectional crosstalk between the AD brain and the periphery. Despite that the actual source of circulating cytokines has yet to be completely elucidated, assessing their peripheral profile could help shed light on the evolution of the inflammatory response throughout AD progression. To date, the results on circulating cytokines in AD patients are controversial—probably on account of the different methodological approaches used for their peripheral detection.

#### 4.1.1. Pro-Inflammatory Cytokines

IL-6, IL-1β and TNF-α can be considered among the principal mediators of the proinflammatory response in AD. In the CNS, these inflammatory mediators are released as a result of glial exposure to Aβ [43,99], while, in the periphery, they are commonly produced by monocytes/macrophages during the acute phase of the immune response [100]. Both increased [101,102,103,104] and unchanged [105,106,107] peripheral levels of those proinflammatory cytokines have been reported in AD patients compared to healthy controls. On the other hand, the concentrations of IL-6 and IL-1β were found significantly lower in MCI patients compared to AD ones [102,108], while the TNF-α concentration appeared to be disease stage-dependent, with decreased levels in patients with mild-to-moderate AD compared to those with severe AD [109]. Finally, a significant correlation was observed in AD patients between the IL-6 levels in matched CSF and blood samples [110].

#### 4.1.2. Anti-Inflammatory Cytokines

IL-10, IL-4 and TGF-β may be regarded as the anti-inflammatory cytokines putatively able to limit proinflammatory activation in AD. Their main activities concern the suppression of macrophage activation and the reduction of proinflammatory cytokine synthesis [111,112,113,114]; moreover, while IL-10 and IL-4 inhibit Th1 lymphocytes while fostering a Th2 response—with a subsequent induction of IgG secretion by B cells [115,116], TGF-β acts as a potent suppressor of both Th1 and Th2 cells [117]. In the CNS, IL-10 acts as a modulator of glial activation; it mediates the crosstalk between the microglia and astrocytes and inhibits the excessive production of proinflammatory mediators, thereby exerting neuroprotective effects [118]. IL-4 exerts a neuroprotective effect by eliciting neuroprotective phenotypes in astrocytes and the microglia, with critical effects on higher brain functions, such as learning and memory [119,120,121]. Finally, TGF-β promotes neuroprotection by means of microglia-mediated Aβ degradation [122]. Studies evaluating the levels of IL-10 and IL-4 in the periphery of patients with AD are not consistent; however, unaltered levels—compared to healthy controls—have been reported in most studies [123,124,125]. On the other hand, AD patients showed significantly elevated TGF-β peripheral levels [126,127], with higher concentrations in patients with mild-to-moderate AD and lower concentrations in patients with severe AD [128].

### 4.2. Chemokines as Biomarkers for Peripheral Immune Cells Recruitment

Chemokines are chemotactic cytokines essential for the activation and migration of specific subsets of leukocytes in order to elicit targeted and specialized immune responses. In AD, the development of neuroinflammation is accompanied by a generalized upregulation of plaque-associated chemokines and chemokine receptors, leading to peripheral monocyte recruitment and glial cell activation [62,129].

#### 4.2.1. Monocyte Chemotactic Protein 1 (MCP-1)

Monocyte chemotactic protein 1 (MCP-1)—also known as CC chemokine ligand 2 (CCL2)—was the first chemokine associated with AD and plays a pivotal role in the recruitment and accumulation of immune cells at the level of senile plaques. The inflammatory responses mediated by MCP-1 are linked to Aβ pathology, as the Aβ-induced upregulation of MCP-1 has been demonstrated in the microglia, astrocytes and human monocytes [130,131]. It is plausible that plaque-associated MCP1 production is related to an initial attempt of glial cells to eliminate Aβ deposits by means of resident and peripheral phagocyte recruitment [132,133]; however, its overexpression in the late stages of AD may induce inflammatory states with detrimental effects on the brain [134]. The clinical data of AD patients have shown an increase of CCL2 both in the CSF and the periphery compared to the healthy controls [135,136] and a strong positive correlation between the matched samples [110]. In particular, in line with the hypothesized role in the early stage of the disease, higher MCP-1 concentrations were found, especially in MCI patients [137].

Despite most chemokines contributing to AD by recruiting peripheral immune cells and promoting glial activation, emerging data hypothesize for some of them an additional anti-inflammatory and neuroprotective role.

#### 4.2.2. CXCL8/IL-8

Interleukin 8 (IL-8)—also known as CXCL8—is a chemokine produced in response to Aβ and proinflammatory stimuli, both in the CNS and the periphery [138,139], and has been found to be increased in the blood and CSF of AD patients [140,141]. Together with its principal function of neutrophils and T-lymphocyte recruitment [142,143], CXCL8 also shows a neuroprotective effect by inhibiting Aβ-induced apoptosis and upregulating the brain-derived neurotrophic factor (BDNF) [144].

#### 4.2.3. CX3CL1 (Fractalkine)

Soluble Fractalkine is a chemoattractant for NK cells predominantly expressed in the CNS by neurons and glial cells [145]. It appears that this chemokine is also able to exert an anti-inflammatory function by mediating the neuronal regulation of microglial activation and overproduction of inflammatory mediators, thereby preventing neurotoxicity [146]. The central and peripheral levels of Fractalkine have been reported to be significantly elevated in patients with MCI compared to patients with severe AD [147,148], supporting the idea that, during AD pathogenesis, Fractalkine may lose control over microglial activation, paving the way to neurodegeneration.

### 4.3. YKL-40 as Biomarker for Astrocytes (and Macrophages) Activation

YKL-40, also known as chitinase 3-like protein-1 (CHI3L1), is a secreted glycoprotein belonging to the human chitinase family, named after its three N-terminal amino acids—tyrosine (Y), lysine (K) and leucine (L)—and its molecular weight of 40 kDa [149]. YKL-40 is expressed in a wide variety of tissues, including the CNS, and is upregulated in a number of inflammatory conditions in response to the proinflammatory cytokines TNF-α and IL-1β [150,151]. In the periphery, YKL-40 is expressed principally by activated macrophages and exerts a significant impact on their alternative (M2) differentiation [152,153]. The pattern of YKL-40 expression in the CNS has long been a subject of debate; despite being initially associated with the macrophages/microglia lineage, evidence suggests that, during neuroinflammatory processes, its synthesis is enhanced predominantly in reactive astrocytes and only occasionally in the microglia [154].

In the AD brain, YKL-40 is expressed by reactive astrocytes around amyloid plaques and blood vessels with CAA [155,156]. It has been suggested that YKL-40 transcription is induced in astrocytes by proinflammatory cytokines released from macrophages, as a result of the inflammatory processes taking place in the CNS or in the periphery [151]. Despite that the precise function of YKL-40 in AD remains to be elucidated, it seems to be involved in Aβ phagocytosis and amyloid plaque formation, with a still undetermined beneficial or detrimental role [157,158].

Since the very first analysis carried out by Craig-Schapiro, several studies have consistently demonstrated increased CSF levels of YKL-40 in patients with AD compared with healthy controls [155,159]; in particular, the CSF YKL-40 level is already increased during the preclinical and MCI phases of AD [160]—a hint that immune system activation occurs early in the disease—and increases longitudinally over time along the AD continuum [161]. Furthermore, higher baseline levels of CSF YKL-40 in preclinical and MCI patients are associated with an increased risk of progression to AD [161]. Similarly, the plasma levels of YKL-40 are elevated in AD patients compared to the MCI and controls; however, mild AD patients show significantly higher levels compared to moderate/severe AD [162]. In addition, plasma YKL-40 does not seem to demonstrate a prognostic utility for cognitive decline [155].

### 4.4. TREM2 as Biomarker of Phagocytic Activity

**Figure 2:**
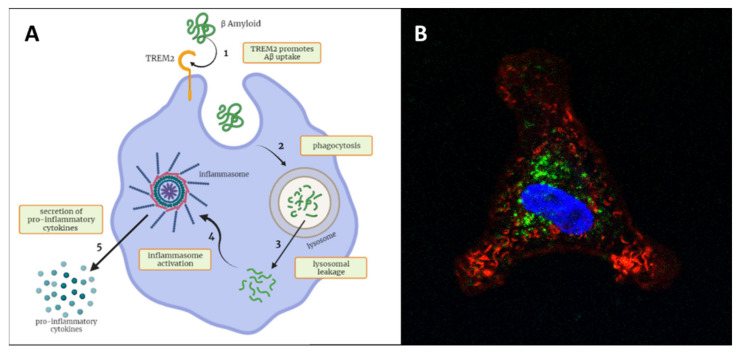
(**A**) Mononucleate cells phagocytize beta-amyloid by TREM2 involvement (1). Due to the beta sheet-rich structure, the autophagy–lysosomal system (2) eventually results in incompetence for a full degradation and (3) leakage of the toxic peptide activates the inflammasome (4) with the secretion of proinflammatory cytokines (5), fueling downstream neuroinflammation. (**B**) In vitro phagocytosis assay; fluorescence micrograph showing beta-amyloid (in green) phagocytized by a THP-1 cell (acute monocytic leukemia line) (courtesy of Virginia Rodriguez-Menendez, UNIMIB, Italy). Created with BioRender.com.

The Triggering receptor expressed on myeloid cells 2 (TREM2) is a transmembrane receptor expressed on the cell surface of myeloid cells (i.e., monocytes and macrophages)—including microglial cells in the CNS [163,164]—and involved in the regulation of phagocytosis [165]. The increased expression of TREM2 has been confirmed in peripheral monocytes of AD patients compared to controls [166] and of MCI patients progressing to AD (converter) compared to MCI nonconverters and to AD patients [167]. In the AD brain, TREM2 is expressed by amyloid-associated microglia and peripherally recruited monocytes/macrophages [168,169,170]; upon binding oligomeric Aβ [171], it mediates the myeloid cell recruitment and accumulation around plaques [169,172], as well as amyloid uptake and degradation [173,174] (Figure 2). In light of these premises, it is possible to speculate a protective role of TREM2 in the early stages of AD.

Moreover, TREM2 undergoes proteolytic cleavage and is released as a soluble form (sTREM2) [175]. The sTREM2 CSF levels are significantly elevated in both MCI and AD patients compared to healthy controls [176,177] and change in a disease stage–dependent manner, peaking at the early symptomatic phase of the disease and decreasing during AD progression [178,179]. The existing studies have generally reported no significant difference in the plasma levels of sTREM2 between AD patients and healthy controls [179,180]; however, a positive correlation was observed between the CSF and plasma sTREM2 levels [177]. Accumulating evidence suggests that sTREM2 is able to exert a neuroprotective effect [181] and possesses many functional aspects resembling those of the full-length membrane-bound protein, including the specific interaction with oligomeric Aβ and subsequent reduction of amyloid deposition by means of microglial recruitment and Aβ uptake and degradation [182].

### 4.5. TSPO as Classic Biomarker of Neuroinflammation with a Putative Role in Peripheral Monocytes Recruitment

The translocator protein 18 kDa (TSPO) is a transmembrane protein widely expressed throughout the body originally discovered as a peripheral binding site for benzodiazepines [183]. Although TSPO is primarily located on the outer mitochondrial membrane, it has also been identified on the plasma membrane of various cell types, including glia and monocytes/macrophages [64,184,185]. In the CNS under physiological conditions, the TSPO levels are very low; however, they experience a dramatic increase in response to neuroinflammation as a result of glial activation and peripheral monocyte recruitment [186]. Its marked upregulation in a broad spectrum of neuropathological conditions has made TSPO a leading biomarker of neuroinflammation. To this end, the TSPO levels can be assessed using in vitro and in vivo imaging techniques with high selective tracers [187].

AD and MCI patients have been found to show significantly increased TSPO expression compared with age-matched healthy controls [188]. In particular, the TSPO levels seem to increase in glial cells before the appearance of AD-associated brain lesions and, better yet, predict the subsequent neurodegeneration in the same areas [189]. Furthermore, longitudinal studies suggest that TSPO levels increase over time throughout AD progression from MCI to full-blown dementia [190] and that astrocytes upregulate TSPO earlier in AD pathology compared to the microglia [191].

Despite its wide employment in imaging studies, the precise contribution of TSPO to the neuroinflammation in AD remains unclear. Of note, TSPO ligands have been shown to modulate chemotaxis in peripheral monocytes [65], and diazepam-binding inhibitor (DBI)—the endogenous ligand for TSPO—has been found upregulated in the CSF and serum of AD patients [192,193]. Intriguingly, serum DBI displayed a further increase in patients with delirium [193]. In light of the aforementioned common pathogenic mechanism (neuroinflammation) between delirium and AD, it is possible to speculate on a possible involvement of the DBI/TSPO system in peripheral monocyte recruitment in the CNS (Figure 1).

## 5. Conclusions

Neuroinflammation is a central feature in the development of AD—contributing to its pathogenesis just as much as Aβ plaques and NFT—and this may be especially relevant when thinking of the partial failure of anti-amyloid approaches. Apart from merely mirroring the pathogenic changes happening into the AD CNS, the peripheral immune system contributes to neuroinflammation by means of phagocytic cells and inflammatory mediators able to access the brain parenchyma through a functionally impaired BBB. According to this vision, peripheral biomarkers of neuroinflammation should be considered as true *core markers* of AD, accessible for implementing the diagnosis and the clinical follow-up of patients. Further studies with novel sensitive techniques (e.g., Simoa) comparing the central and peripheral compartments are therefore advisable, analogously to what has been recently made, for example, for phosphorylated tau [194].

Finally, effective disease-modifying therapies for AD are still a substantially unmet need, and focusing on the dysregulated peripheral inflammatory responses represents an interesting line of research. As a matter of fact, modulating cytokine/chemokine production, monocyte chemotaxis and neuro-invasion and Aβ phagocytosis could eventually slow down AD-related neurodegeneration. Theoretically, we may even think to target neuroinflammation right from the periphery by means of drugs that do not cross the BBB (e.g., Etanercept [195]). Last, but not least, in light of the clinical and pathogenic liaisons with the neurobehavioral syndrome of delirium, targeting peripheral inflammatory processes could also be interesting for specifically treating the behavioral symptoms of AD [196]. At the moment, clinical studies are required to clarify if drugs able to modulate these processes may eventually succeed in mitigating the clinical phenotype and delaying the conversion in prodromal AD patients.
